# Assessment of anxiety in children with neurodevelopment disorders: Rasch analysis of the Spence Children’s Anxiety Scale

**DOI:** 10.3389/fpsyt.2024.1240357

**Published:** 2024-04-29

**Authors:** Alana Sparks, Susan Gilbert Evans, Mojib Javadi, Bianca Lasalandra, Emily Martens, Raadhika Venkatesh, Izzy T. Vaccarino, Anthony L. Vaccarino

**Affiliations:** Indoc Research, Toronto, ON, Canada

**Keywords:** ADHD (attention-deficit/hyperactivity disorder), autism spectrum disorder, anxiety, Rasch analyses, validity, rating scale, neurodevelopmental disorders

## Abstract

Anxiety is common in neurodevelopmental disorders (NDD). The parent version of the Spence Children’s Anxiety Scale (SCAS-P) is a widely used measure to assess anxiety across a broad range of childhood populations. However, assessment of the measurement properties of the SCAS-P in NDDs have been limited. The present study aimed to assess the psychometric properties of the SCAS-P in children with attention-deficit/hyperactivity disorder (ADHD) and autism spectrum disorder (ASD) using Rasch Measurement Theory. Data from the Province of Ontario Neurodevelopmental Disorders Network Registry were used in the analysis. Children (ages 6-13 years old) with a primary diagnosis of ADHD (n=146) or ASD (n=104) were administered the SCAS-P. Rasch Measurement Theory was used to assess measurement properties of the SCAS-P, including unidimensionality and item-level fit, category ordering, item targeting, person separation index and reliability and differential item functioning. The SCAS-P fit well to the Rasch model in both ADHD and ASD, including unidimensionality, satisfactory category ordering and goodness-of-fit. However, item-person measures showed poor precision at lower levels of anxiety. Some items showed differential item functioning, including items within the obsessive-compulsive, panic/agoraphobia and physical injury fears domains, suggesting that the presentation of anxiety may differ between ADHD and ASD. Overall, the results generally support the use of the SCAS-P to screen and monitor anxiety symptoms in children with ADHD and ASD. Future studies would benefit from examination of more severely anxious NDD cohort, including those with clinically diagnosed anxiety.

## Introduction

Anxiety is a common mental health problem in children with attention-deficit/hyperactivity disorder (ADHD) (1–3) and 40% of children with autism spectrum disorder (ASD) (4, 5). Comorbid anxiety in children with NDDs can cause distress and is associated impaired functioning (6, 7). Accurate assessments of anxiety are therefore needed to identify and monitor symptoms of anxiety in children living with NDDs. However, there are challenges to assessing anxiety in children with NDDs, including overlapping features of NDDs and anxiety that can confound the assessment of anxiety and lead to misinterpretation of anxiety-related signs and symptoms (8, 9). Indeed, there is considerable overlap in diagnostic symptom criteria for ADHD and anxiety, including restlessness and distractibility (8). In ASD, deficits in social interactions and stereotyped, repetitive motor movements too can be misinterpreted as anxiety and vice versa (9). These challenges are particularly difficult given co-occurring intellectual disabilities and language impairments (9).

A number of scales have been developed to assess symptoms of anxiety in children, including the Revised Children’s Anxiety and Depression Scale (10), the Screen for Child Anxiety Related Emotional Disorders (11) and the Spence Children’s Anxiety Scale (SCAS) (12). However, most scales that are used to assess anxiety in NDDs were developed and validated in typically developing children and validation studies in children with NDDs have been limited (9). The SCAS was developed to assess the severity of anxiety symptoms in children that is based on six anxiety disorder dimensions, including generalized anxiety, panic, social phobia, separation anxiety, obsessive compulsive disorder and fear of injuries (12, 13). The SCAS, therefore, can be a useful tool to assess and monitor anxiety in children and has been used across a broad range of childhood populations, including children with NDDs (14). In children with ASD, the SCAS was found to have good psychometric properties (14–17). However, differences in factor structure of the SCAS between anxious children with or without ASD diagnosis have been noted, suggesting presence of ASD-related signs and symptoms may impact the presentation of anxiety (14, 16, 18). The validity of the SCAS in children with ADHD has not been addressed.

Given the high rates of comorbid anxiety and inherent challenges in assessing anxiety in NDD, is important that the psychometric properties of the SCAS be further evaluated in children with NDDs. The objective of this study was to assess the psychometric properties of the parent version of the SCAS (SCAS-P) in children with ADHD and ASD using Rasch Measurement Theory (RMT) (19, 20). RMT considers the probability of an item’s score as a function of both the person’s individual trait level (i.e., level of anxiety) and the item’s difficulty (i.e., level of anxiety that item assesses) (19). In this context, children with higher levels of anxiety have a greater chance of endorsing an anxiety item, and an item that assesses lower levels of anxiety are more likely to be endorsed than items that assess higher levels of anxiety. RMT provides fundamental criteria for objective scale measurement and determines how well the observed data approximates the Rasch measurement model. Items that do not fit the model are indication that they may be measuring more than one construct, thus calling into question the scales construct validity. RMT approach, therefore, can be particularly useful in evaluating the construct validity and generalizability of rating scales in populations that they were not originally developed. We recently used this approach to evaluate depressive symptoms in adults with neurological disorders, where the overlap between symptoms of depression and neurological disorders could potentially confound the assessment of depression (21). The same approach was used in the present study, including Rasch-based criteria to assess item-level goodness of fit, category ordering, item targeting, person separation index and reliability and item bias (differential item functioning) (21–23).

## Methods

### Study population

The present study used de-identified data from the Province of Ontario Neurodevelopmental Disorders Network (POND) Registry; a multi-centre research network studying the neurobiology of NDDs (24). These data are currently stored in the Brain-CODE Neuroinformatics Platform (25) and were made available for secondary use through a controlled public data release from the Ontario Brain Institute (www.braincode.ca). Data included demographic and SCAS-P assessments for children aged 6-13 years old, with a primary diagnosis of ADHD (n=146) or ASD (n=104), as well as small cohort of typically developing (TD) children (n=13). Please see (20) for POND protocol details.

### Assessments

The SCAS-P is a 38-item parent-report measure that assesses the severity of anxiety-related symptoms in children (12, 26). The scale is aligned with symptom domains based on DSM-IV criteria for anxiety disorder, including separation anxiety (6 items), social phobia (6 items), obsessive-compulsive (6 items), panic (6 items)/agoraphobia (3 items), physical injury fears (5 items) and generalized anxiety (6 items). Items are scored on a 4-point scale: 0 = never, 1 = sometimes, 2 = often and 3 = always; a total score is calculated (maximum = 114), as well as for each of the six domains/subscales, with higher scores indicating greater severity of anxiety symptoms.

### Analyses

Demographic and clinical characteristics were calculated and compared across cohorts; ANOVA was used for comparison of continuous variables (age) and chi-squared for comparison of categorical variables (sex). Analyses were performed using SPSS V27. A level of p < 0.05 was regarded as statistically significant. RMT was used to assess performance of the SCAS-P in NDD. Rasch analyses were carried out using WinSteps Version 5.1.

Unidimensionality was examined by principal component analysis of the residuals derived from the Rasch model. The scale was considered unidimensional if > 40% of variance was explained by the measurement variable and unexplained variance of the first contrast accounts for < 10 % (27–29).

Item-level goodness of fit statistics were calculated as an index of how much the observed score for a given item within the scale deviates from the expected score of the Rasch model. Items that did not fit the Rasch model (misfits) do not contribute to measurement of the underlying construct and likely add unwanted noise to the scale. Item “infit” mean square (MNSQ) values provide a fit index for each item that are in close proximity to the person’s severity level, and “outfit” MNSQ values for differences between observed and expected values for items that are far from the person’s severity level. MNSQ values between 0.5 and 1.5 were considered to be acceptable fit, with values between 1.5 and 2 considered to underfit the model, but do not distort the results, and values greater than 2.0 flagged as misfits that can distort the scale (30).

Category threshold ordering determined whether participants can discriminate between the ordered response options (i.e., never, sometimes, often, always) with fit values between 0.5 and 1.5 considered to be acceptable fit, and values greater than 2.0 flagged as misfitting (30).

Person-item maps plot individual participants and items on a single continuum to compare the range and position of the person measure distribution to that of the item measure distribution. Both item “difficulty” (i.e., the level of anxiety that item assesses) and person “ability” (i.e., level of symptom severity) are visualized together on a logit scale (i.e., log of the odds); with the right side of the map displaying the items from most difficult (top) to least difficult (bottom) and the left side plotting the individual participants, with those at the top having the highest trait level (symptom severity) and those at the bottom the least. The targeting of the scale is assessed by comparing mean person and mean item logit locations, with good measurement targeting evidenced when mean persons and items that are in close proximity to one another (within 1 logit) (31, 32). The clinical utility of summing individual items from a scale to form a total score of overall severity requires that the items be spread out across the severity level of a broad range of persons. Gaps between items impact the scale’s sensitivity, as persons falling within those gaps cannot be differentiated from one another.

To assess potential item biases, differential item functioning (DIF) was used to determine whether items show differences in item difficulty between groups (i.e., whether subgroups with similar levels of anxiety have the same probability of endorsing a given item). In the present study, DIF are indications that the expression of anxiety may differ between subgroups. The existence of DIF was assessed by cohort (ADHD vs ASD) and sex (male vs female), with mean differences in person measures > 0.64 logits (with p<0.05 in Rasch-Welch test statistic) as indications of significant and meaningful DIF (31, 32).

Reliability of the SCAS-P was evaluated using item and person separation indices and reliability derived from the Rasch model (30). The person separation index provides an estimate of spread of participants that reflect the number of distinct levels of severity that can be distinguished (strata), with person separation indices of > 1.5 considered acceptable(minimum required to divide sample into two distinct strata). Person reliability is analogous to Cronbach’s alpha as a measure of internal constancy, with values of >0.70 as indication of acceptable internal consistency. That is, confirmation that a person with higher levels of the underlying trait do indeed score higher on the scale than those with lower levels of the underlying trait, and vice versa. The item separation index was used to confirm hierarchy of items, with item separation indices of > 2 and reliability >0.8 considered acceptable to support the scales construct validity (30).

## Results

Demographic and clinical characteristics are shown in **Table 1**. The ADHD and ASD cohorts were predominately male (79.45 and 84.62%, respectively) and that is consistent with the higher rates reported by males (33, 34). The ADHD cohort reported greater *social phobia* than ASD, whereas the ASD cohort reported greater *obsessive-compulsive behaviors, panic/agoraphobia* and *fears of physical injury* than the ADHD cohort (see **Table 1**). SCAS-P total scores did not differ between NDD cohorts. Demographic and clinical characteristics for the TD cohort are also shown in **Table 1** for comparison (statistical comparisons to the TD cohort were not reported given the small sample size).

**Table 1:** Demographic and clinical characteristics.

	ADHD	ASD	TD
**N**	146	104	13
**AGE, YEARS ± SD**	7.42 ± 1.19	7.36 ± 1.33	7.39 ± 1.22
**SEX, % FEMALE**	20.55%	15.38%	38.46%
**SCAS-TOTAL**	20.15 ± 14.17	21.57 ± 17.11	9.73 ± 6.58
**Separation Anxiety**	5.37 ± 4.00	5.17 ± 4.28	2.36 ± 1.80
**Social Phobia**	4.44 ± 3.74*	3.09 ± 3.71	2.91 ± 2.66
**Obsessive-Compulsive**	1.63 ± 1.84	2.62 ± 3.03*	0.73 ± 1.10
**Panic/agoraphobia**	1.36 ± 2.28	2.82 ± 3.58*	0.09 ± 0.31
**Fears of Physical Injury**	3.43 ± 2.56	4.27 ± 3.13*	1.73 ± 1.90
**Generalized Anxiety**	3.99 ± 3.17	3.73 ± 3.37	1.58 ± 3.21

ADHD, attention-deficit/hyperactivity disorder; ASD, autism spectrum disorder; TD, typically developing; *p<0.05 ADHD vs ASD.

Principal component analysis of the residuals revealed unidimensionality of the SCAS-P that supported a single dominant factor (29, 30), with variances explained by the measurement variables of 48.6% and 40.7% and unexplained variance of the first residual of 4.3% and 5.9% in ADHD and ASD cohorts, respectively.

Item difficulty estimates and fit statistics are shown in **Table 2**. In both cohorts, Item 5 (“My child would feel afraid of being on his/her own at home”) was the least difficult/most endorsed item (ADHD: -2.07 logits; ASD: -1.63 logits) and item 30 (“My child suddenly becoming faint or dizzy for no reason”) the most difficult/least endorsed item (ADHD: 2.51 logits; ASD: 1.99 logits). Most items showed acceptable goodness-of-fit statistics, although some underfitting was noted (MNSQs >1.5, see **Table 2**). In particular, item 16 (“My child is scared of dogs”) was identified as a possible misfit item in the ADHD cohort with infit MNSQ = 1.77 and outfit MNSQ = 2.06, thus questioning the construct validity of this item as a measure of anxiety. This is not surprising given the potential positive impact child-dog interactions can have on wellbeing and can serve as a source of reduced stress (35).

**Table 2:** Item difficulty and fit statistics.

Item description	Domain	ADHD			ASD		
		Item Difficulty	Infit MNSQ	Outfit MNSQ	Item Difficulty	Infit MNSQ	Outfit MNSQ
1…worry about things	*GA*	-1.73	0.36	0.41	-1.18	0.50	0.56
2…scared of dark	*PI*	-1.89	1.04	1.09	-1.16	0.96	0.95
3…funny feeling in stomach	*GA*	-1.10	0.98	1.00	-0.15	0.85	0.79
4…complains of feeling afraid	*GA*	-1.17	0.54	0.59	-0.75	0.51	0.51
5…afraid of being on own at home	*SA*	-2.07	1.52	1.47	-1.28	1.23	1.14
6…scared to take a test	*SP*	-0.86	1.01	0.94	-0.26	0.97	0.87
7…afraid to use public toilets or bathrooms	*SP*	0.04	1.23	1.03	-0.02	1.37	1.26
8…worries about being away	SA	-1.49	0.69	0.79	-1.16	0.84	1.02
9…afraid will make a fool in front of people	SP	-0.96	0.95	0.96	0.12	0.97	0.70
10…worries that will do badly at school	SP	-1.33	0.98	1.03	-0.13	0.94	0.81
11…worries something awful will happen to family	SA	-0.76	0.77	0.74	0.21	1.21	0.81
12…feeling can**’**t breathe when there is no reason	PA	1.10	0.98	0.66	1.86	1.64	0.67
13…has to keep checking that done things right	OC	1.25	1.10	1.16	0.41	0.84	1.29
14…scared to sleep on own	SA	-1.21	1.49	1.52	-0.76	1.48	1.54
15…trouble going to school **…** feels nervous/afraid.	SA	-0.21	0.99	0.86	-0.29	0.96	0.75
16…scared of dogs	PI	0.06	1.77	2.06	-0.53	1.48	1.58
17…can**’**t get bad/silly thoughts out of head	OC	-0.83	0.88	1.09	-0.44	0.90	0.80
18…when there**’**s a problem, heart beating really fast	GA	0.42	0.95	0.78	0.73	0.86	0.52
19…tremble or shake when there is no reason	PA	1.61	1.15	1.10	1.01	1.25	0.88
20…worries something bad will happen to him/her	GA	-0.22	0.71	0.64	0.06	0.76	0.64
21…scared of going to doctor or dentist	PI	-0.54	1.40	2.03	-1.23	1.32	1.56
22…feels shaky when has a problem	GA	1.15	1.03	0.55	0.34	0.88	1.12
23…scared of heights	PI	-0.13	1.39	1.22	-0.27	1.12	1.05
24… think special thoughts to stop bad things	OC	2.12	1.23	0.70	1.12	1.05	0.49
25…scared to travel in car, bus or train	PA	1.84	0.89	0.51	1.30	1.27	1.45
26…worries what other people think of him/her	SP	-0.99	0.92	0.87	0.03	1.06	0.73
27…afraid of being in crowded places	PA	0.66	0.90	0.72	-0.75	0.94	0.89
28…feels really scared for no reason	PA	0.70	0.74	0.52	0.02	0.97	0.88
29…scared of spiders and insects	PI	-0.65	1.50	1.53	-0.31	1.49	1.37
30…suddenly becoming faint or dizzy for no reason	PA	2.51	1.05	0.65	1.59	0.75	0.53
31…afraid to talk in front of class	SP	-0.83	1.24	1.18	-0.32	1.28	1.26
32…heart suddenly beat quick for no reason	PA	1.36	1.18	0.67	1.51	1.09	0.58
33…scared feeling when nothing to be afraid of	PA	0.81	0.94	0.57	0.53	1.04	0.74
34…afraid of being in small spaces	PA	0.85	1.05	0.74	0.19	1.27	1.06
35…has to do some things over and over	OC	0.97	1.09	1.18	-0.23	1.05	1.88
36… bothered by bad/silly thoughts/pictures in head	OC	-0.13	1.04	0.90	-0.04	1.21	1.02
37…has to do certain things right to stop bad things	OC	2.37	1.35	1.85	0.83	1.18	0.83
38…scared to stay away from home overnight	SA	-0.72	1.37	1.16	-0.61	1.24	1.49

GA, generalized anxiety; OC, obsessive-compulsive; PA, panic/agoraphobia; PI, physical injury fears; SA, separation anxiety; SP, social phobia; ADHD, attention-deficit/hyperactivity disorder; ASD, autism spectrum disorder; MNSQ, mean square index.

The category thresholds were ordered indicating that the SCAS-P scale options functioned in sequential order in capturing increases levels of severity, with children with higher levels of anxiety endorsing higher SCAS-P options. Average measures increased in scale categories from -2.74 logits (“never”) to 0.08 logits (“always”) in the ADHD cohort and from -2.33 logits (“never”) to 0.42 logits (“always”) in the ASD cohort. All infit/outfit MNSQ were acceptable (MNSQ < 1.5).

**Figure 1:**
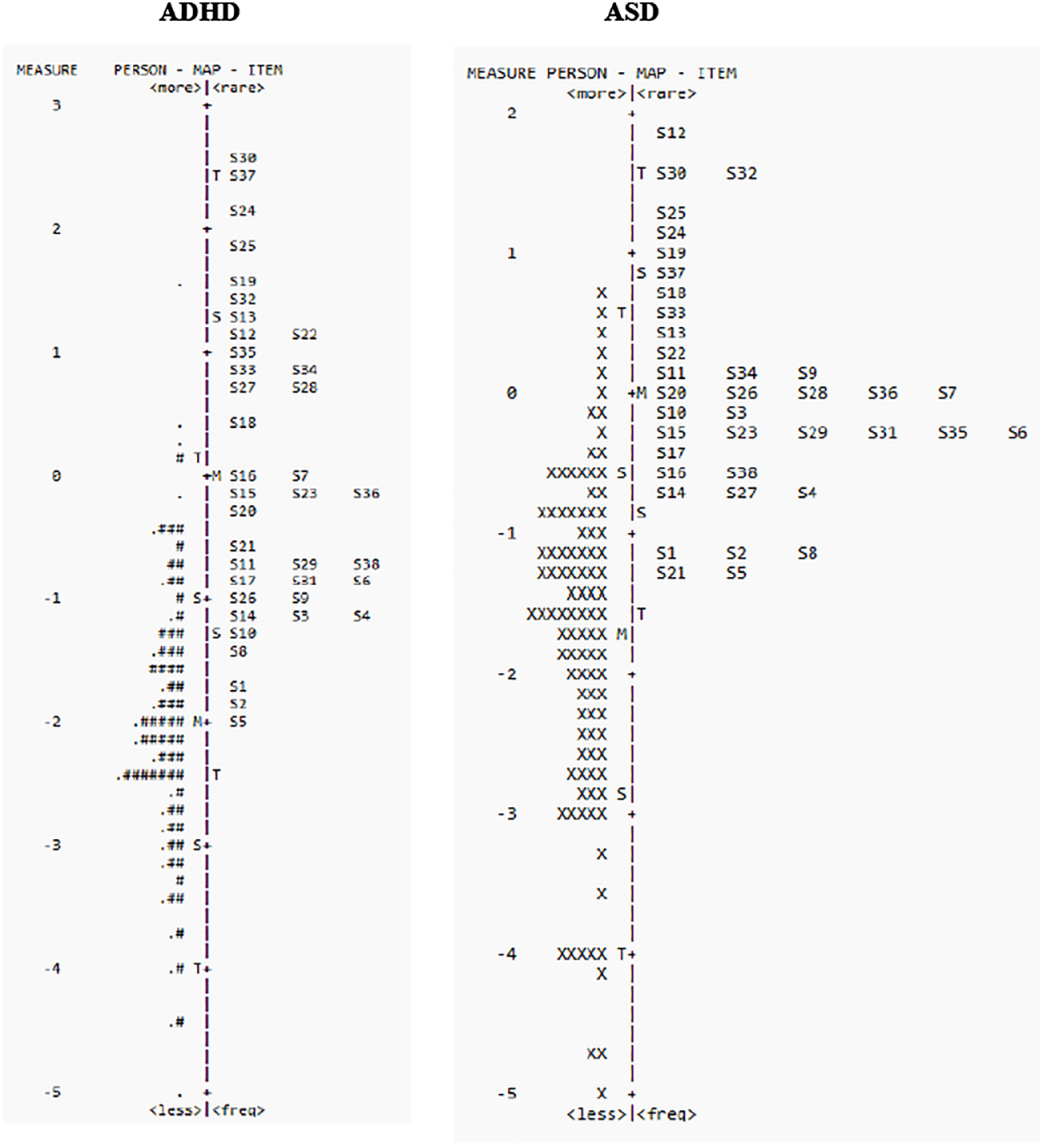
Person-item location (Wright maps) for SCAS-P items in ADHD (left panel) and ASD (right panel). The right side of the map displays the items from most difficult (top) to least difficult (bottom), and the left side plots the individual participants, with those at the top having the highest trait level (depression) and those that the bottom the least. M, mean difficulty; S, one standard deviation; T, two standard deviations.

Person-item locations (Wright maps) for the SCAS are shown in **Figure 1**. The mean person measures were -2.00 and -1.71 logits in ADHD and ASD cohorts, respectively. Therefore, although the SCAS had a wide range of items and no large gaps were noted between items, no items targeted children with anxiety levels below -2.07 logits in ADHD and -1.63 logits in ASD (see **Figure 1**, **Table 2**).

Meaningful DIF (>0.64 logits) between ASD and ADHD was noted for 15 of the 39 items, including multiple items within the “obsessive-compulsive,” “panic-agoraphobia” and “physical injury fears” domains more likely to be endorsed in the ASD than the ADHD cohort, and items within the “social phobia” domain more likely to be endorsed in the ADHD than the ASD cohort (**Table 3**). No DIF was noted based on sex.

**Table 3:** Differential item functioning.

Domain	#	question	DIF contrast	direction
**OC**	13	My child has to keep checking that (s)he has done things right	0.91	ASD > ADHD
**OC**	24	My child has to think special thoughts (like numbers or words) to stop bad things from happening	1.03	ASD > ADHD
**OC**	35	My child has to do some things over and over again	1.29	ASD > ADHD
**OC**	37	My child has to do certain things in just the right way to stop bad things from happening	1.57	ASD > ADHD
**PA**	27	My child is afraid of being in crowded places	1.54	ASD > ADHD
**PA**	28	All of a sudden my child feels really scared for no reason at all.	0.78	ASD > ADHD
**PA**	34	My child is afraid of being in small closed places, like tunnels or small rooms	0.73	ASD > ADHD
**PI**	16	My child is scared of dogs	0.75	ASD > ADHD
**PI**	21	My child is scared of going to the doctor or dentist	0.90	ASD > ADHD
**GA**	22	When my child has a problem, (s)he feels shaky	0.88	ASD > ADHD
**GA**	3	When my child has a problem, s(he) complains of having a funny feeling in his/her stomach	-0.71	ADHD > ASD
**SP**	9	My child feels afraid that (s)he will make a fool of him/herself in front of people	-0.85	ADHD > ASD
**SP**	10	My child worries that (s)he will do badly at school	-0.94	ADHD > ASD
**SP**	26	My child worries what other people think of him/her	-0.80	ADHD > ASD
**SA**	11	My child worries that something awful will happen to someone in our family	-0.77	ADHD > ASD

OC, obsessive-compulsive; PA, panic/agoraphobia; PI, physical injury fears; SA, separation anxiety; SP, social phobia; ADHD, attention-deficit/hyperactivity disorder; ASD, autism spectrum disorder.

Person separation indices and reliability were acceptable in both cohorts, indicating the SCAS-P could stratify NDD participants into at least 3 separate groups, with person separation indices and reliability of 2.83 and 0.89 in the ADHD cohort, respectively and 2.73 and 0.88 in the ASD cohort, respectively. Internal consistency was also good, with Cronbach’s alpha of 0.97 (ADHD) and 0.94 (ASD), consistent with previous reports in ASD of 0.94 (15). The scales construct validity and hierarchy of items was also supported, with acceptable item separation indices and reliability of 6.07 and 0.97 for ADHD and 4.08 and 0.94 for ASD, respectively.

## Discussion

The present study used RMT to evaluate the psychometric properties of the SCAS-P in children with ADHD and ASD. The results generally support the use of the SCAS-P to assess anxiety in children with ADHD and ASD, including unidimensionality and acceptable item-level goodness-of-fit statistics, suggesting that all items contributed to the same underlying construct. However, item difficulty estimates and person-item maps showed poor item targeting at lower levels anxiety (see **Figure 1**). This is not surprising as POND participants were recruited based on primary NDD diagnosis and not presence (or absence) of comorbid anxiety (24). None-the-less, person separation indices and reliability indicated that in the present cohort the SCAS-P can effectively discriminate different levels of anxiety and can therefore be a useful tool screen and monitor anxiety in children with NDDs.

The present study also compared the presentation of anxiety-related symptoms between ADHD and ASD cohorts. DIF revealed that children with ASD were more likely to endorse anxiety symptoms related to *OC behaviours*, *panic/agoraphobia* and *physical fear-related behaviours*, than the ADHD cohort (see **Table 3**). These results are consistent with those reported by Toscano et al. (18) that children with ASD and comorbid anxiety show higher levels of anxiety related to *fears of physical injuries* and *OC behaviours*, as compared to children with anxiety without an ASD diagnosis. On the other hand, DIF revealed that children in the ADHD cohort were more likely to endorse anxiety related to *social phobias*, than the ASD cohort, including *afraid that (s)he will make a fool of him/herself in front of people* (item 9), *worries that (s)he will do badly at school* (item 10) and *worries what other people think of him/her* (item 26). Indeed, previous studies have demonstrated that anxiety in children with ADHD is associated with poorer social functioning (36). Taken together, these results suggest the expression of anxiety may be different across NDDs and should be considered in the assessment of anxiety-related behaviours.

Interestingly, DIF revealed that within the OC domain, children with ASD were more likely to express compulsive-like thoughts/behaviours, including *checking that (s)he has done things right* (item 13)*, think special thoughts to stop bad things from happening* (item 24), *do things over and over again* (item 35) and *do certain things in just the right way to stop bad things from happening* (item 37), whereas the cohorts did not differ in obsessive-like thoughts, such as *bad/silly thoughts in head* (items 17 and 36). As obsessions are intrusive, recurring thoughts that cause anxiety and distress, compulsions are in response to these intrusive thoughts that are meant to control anxiety (13). This is consistent with the compulsive behaviors in ASD as being a way of reducing anxiety and managing intrusive thoughts and overwhelming sensory inputs (37). However, although compulsions in OCD are unwanted and distressing, compulsive behaviours in ASD can provide comfort (38). Furthermore, the similarity between ASD-related repetitive behaviours and OCD-related behaviours can make it difficult to differentiate their underlying motivation (37, 38). This should be considered in the assessment of OC behaviours in ASD, as obsessions and compulsions in ASD may have different dynamics than OCD that can lead to misinterpretation of OC features in ASD.

It is important to note that in the present study only the parent version of the SCAS was used. Although previous studies have demonstrated generally good concordance between parent and child versions of the SCAS in typically developing children (26, 39), concordance is reduced in children with ASD (36). Given the intellectual and verbal challenges in children with ASD and other NDDs, it is possible that reduced parent-child concordance is related to the child’s difficulty in communicating anxiety-related thoughts and feelings (39). Indeed, child’s verbal ability is associated with greater parent-child concordance, suggesting that better verbal skills facilitate communication of anxiety-related feelings and reduce ambiguity between anxiety and NDD-related signs and symptoms (39, 40). Moreover, parent-child concordance is higher for observable symptoms, such as separation anxiety, as compared to internalized symptoms, such as generalized anxiety (39, 40). It is possible therefore that compulsive behaviours in ASD are more easily recognized by the parent, than internal obsessive thoughts. In the present study this may have led to a bias towards observable behaviours and underrepresentation of internalized symptoms of anxiety. Assessment of anxiety in children with NDDs based on the SCAS-P therefore should be interpreted with caution, as it relies on interpretation of a child’s behaviour and recognition of internalized symptoms of anxiety. Furthermore, as the SCAS-P is based on the parent’s own conceptualizations of anxiety and interpretation of symptoms, it is important that other sources of information also be considered. To get a more complete picture, assessment of anxiety in children with NDDs would benefit from inclusion of multi-informant information (9, 40).

The POND Network is part of the Ontario Brain Institute’s Integrated Discovery programs (41). These programs generate diverse data types that are integrated within the Brain-CODE platform to support cross-disease comparisons (25). In particular, the establishment of common data elements provides consistency in data collection and optimizes pooling of data and cross-disorder comparisons (42). In the present study, demographic information and SCAS-P were collected consistently across cohorts, thus allowing us to pool and compare ADHD and ASD cohorts. Overall, the present study supports the SCAS-P as a valid instrument to screen and monitor anxiety-related symptoms in children with ADHD and ASD, including children without formally diagnosed anxiety disorders. As a screening tool for comorbid anxiety disorders in children with NDDs, therefore, the SCAS-P can provide important information about the presence of anxiety-related symptoms that may warrant clinical follow-up (9). However, there still remains inherent challenges to assessing anxiety in children with NDDs, including communication difficulties, overlapping features of anxiety and NDD, as well as potential differences in the presentation of anxiety across NDDs (6, 8, 9). These factors will add unwanted noise the measurement tool and reduce its sensitivity to screen and monitor for anxiety.

## Limitations and future directions

As the present study involved secondary use of data, some limitations were noted. SCAS-P items were found to target more severe anxiety than experienced in the present NDD cohorts, which was not surprising as comorbid anxiety was not considered a selection criterion for participation in POND (24). The performance of the SCAS-P in more severely anxious cohorts would require additional studies, including examination in those with clinically diagnosed anxiety. Furthermore, although previous studies have shown that anxiety is a common mental health issue in children with NDDs (1–5), the relatively small sample size of the typically developing children limited comparisons with the ASD and ADHD cohorts. Larger sample size of typically developing children would be needed to support any statistical inferences with respect to the higher levels of anxiety in children with NDDs observed this study.

The present study shows that the SCAS-P can be a valuable tool to assess and monitor anxiety in children with NDD and has implications regarding presentation of anxiety-related symptoms across NDDs and challenges in assessing these symptoms, including confounding NDD-related symptoms, and language and intellectual challenges. Future research should address these challenges, including the modification of existing scales or development of new fit-for-purpose anxiety measures designed specifically for children with NDDs (9). This, of course, will require a better understanding of anxiety-related behaviours in children with NDDs and challenges in assessing those behaviours.

## Data availability statement

The data analyzed in this study is subject to the following licenses/restrictions: Participants’ data used in this study are currently stored in the Brain-CODE Neuroinformatics Platform (https://www.braincode.ca/) managed by the Ontario Brain Institute. Requests to access these datasets should be directed to Ontario Brain Institute at info@braininstitute.ca.

## Ethics statement

The studies involving humans were approved by Canadian SHIELD Ethics Review Board. The studies were conducted in accordance with the local legislation and institutional requirements. Written informed consent for participation in this study was provided by the participants’ legal guardians/next of kin.

## Author contributions

AV and AS: study design and analysis, SE, MJ, BL, EM, AS, RV: data management and curation. AV, AS, IV: manuscript preparation. All authors contributed to the article and approved the submitted version.
